# Decadal stability in genetic variation and structure in the intertidal seaweed *Fucus serratus* (Heterokontophyta: Fucaceae)

**DOI:** 10.1186/s12862-018-1213-2

**Published:** 2018-06-15

**Authors:** Alexander Jueterbock, James A. Coyer, Jeanine L. Olsen, Galice Hoarau

**Affiliations:** 1 Faculty of Biosciences and Aquaculture, Nord University, 8049 Bodø, Norway; 2 Shoals Marine Laboratory, University of New Hampshire, Durham, NH 03824 USA; 3 Ecological Genetics-Genomics Group, Groningen Institute for Evolutionary Life Sciences, University of Groningen, 9747 AG Groningen, The Netherlands

**Keywords:** Brown algae, Effective population size, Evolutionary potential, Genetic diversity, Microsatellites, North Atlantic

## Abstract

**Background:**

The spatial distribution of genetic diversity and structure has important implications for conservation as it reveals a species’ strong and weak points with regard to stability and evolutionary capacity. Temporal genetic stability is rarely tested in marine species other than commercially important fishes, but is crucial for the utility of temporal snapshots in conservation management. High and stable diversity can help to mitigate the predicted northward range shift of seaweeds under the impact of climate change. Given the key ecological role of fucoid seaweeds along rocky shores, the positive effect of genetic diversity may reach beyond the species level to stabilize the entire intertidal ecosystem along the temperate North Atlantic. In this study, we estimated the effective population size, as well as temporal changes in genetic structure and diversity of the seaweed *F. serratus* using 22 microsatellite markers. Samples were taken across latitudes and a range of temperature regimes at seven locations with decadal sampling (2000 and 2010).

**Results:**

Across latitudes, genetic structure and diversity remained stable over 5–10 generations. Stable small-scale structure enhanced regional diversity throughout the species’ range. In accordance with its biogeographic history, effective population size and diversity peaked in the species’ mid-range in Brittany (France), and declined towards its leading and trailing edge to the north and south. At the species’ southern edge, multi-locus-heterozygosity displayed a strong decline from 1999 to 2010.

**Conclusion:**

Temporally stable genetic structure over small spatial scales is a potential driver for local adaptation and species radiation in the genus *Fucus*. Survival and adaptation of the low-diversity leading edge of *F. serratus* may be enhanced by regional gene flow and ‘surfing’ of favorable mutations or impaired by the accumulation of deleterious mutations. Our results have clear implications for the conservation of *F. serratus* at its genetically unique southern edge in Northwest Iberia, where increasing temperatures are likely the major cause for the decline not only of *F. serratus,* but also other intertidal and subtidal macroalgae. We expect that *F. serratus* will disappear from Northwest Iberia by 2100 if genetic rescue is not induced by the influx of genetic variation from Brittany.

**Electronic supplementary material:**

The online version of this article (10.1186/s12862-018-1213-2) contains supplementary material, which is available to authorized users.

## Background

Understanding temporal stability of genetic structure and diversity is crucial for the utility of temporal snapshots in conservation management and to infer how climate-induced range shifts might affect the future distribution and adaptive potential of species. In trailing edge populations, effective population size and genetic diversity are considered major keys to adaptive potential and subsequent persistence under climate change [1, 2]. In contrast, the evolutionary potential and survival of low-diversity leading edge populations [3] may be either enhanced or impaired by the ‘surfing’ of new mutations that can rapidly increase in frequency over iterated founder events, depending on whether the new mutations are primarily favorable or deleterious [4–9].

Studies that assess temporal genetic stability are rare in marine species, and mostly confined to fisheries management to ensure sustainable exploitation of economically important species [10–14]. While high gene flow explained 5 to 24-year long stability in genetic variability and structure in Chinook salmon and Atlantic herring [10, 11], large fluctuations in allele frequencies were recorded over a few months in small and closed populations of the intertidal isopod *Jaera albifrons* [15]. However, high gene flow does not always warrant temporal genetic stability, as several marine species with long-lived planktonic larvae showed stronger temporal than spatial differentiation over 3 to 9 years [16–18]. On the other hand, low gene flow does not necessarily result in genetic instability over time, although genetic drift in small and closed populations can be expected to be high. For example, genetic diversity and population structure remained stable over 5–12 years in relatively closed populations of the seagrass *Zostera marina* [19] and over 2 years in nine out of 10 locally differentiated populations of the isopod *Excirolana braziliensis* [20]. These contrasting results demonstrate that a species’ life history alone does not necessarily predict its genetic stability over time.

Due to their high sensitivity to rising temperatures, responses of marine intertidal species are considered as early warning signals for the impact of climate change [21–25]. Among global climate change factors, ocean warming is considered the most severe threat for marine macrophytes [26–28]. Over the next century, ecological niche models predict the disappearance of intertidal fucoid brown algae along their southern trailing edges and a poleward extension of their northern leading edges [26, 29]. Fucoid brown algae (Heterokontophyta; Fucaceae) are habitat-forming ecosystem engineers supporting species-rich intertidal communities along temperate rocky shores [30–33]. Thus, range shifts of fucoids will undoubtedly trigger major ecological changes along temperate rocky shores of the North Atlantic.

Ecological niche models, however, do not consider the species’ plastic and adaptive potential that could mitigate the predicted northward shifts. Adaptive potential depends largely on a population’s genetically effective size, *N*_*e*_ [34], or the size of an ideal population that undergoes the same rate of genetic change as the real population [35]. At low *N*_*e*_, and low gene flow between populations, genetic drift generally plays the predominant role, effectively neutralizing selection, and eroding genetic diversity through stochastic fixation or loss of allelic variations [36–38]. Although *N*_*e*_ and temporal stability of genetic diversity patterns are particularly important for restoration and conservation efforts of fucoid seaweeds, only a single Norwegian population of *F. serratus* has so far been assessed [39].

The canopy-forming seaweed *F. serratus* is an excellent model for the study of temporal evolution and stability of genetic structure and diversity across a range of contrasting temperature regimes. It is one of the dominant intertidal seaweeds along the Northeast-Atlantic rocky shore from northern Portugal to northern Norway [40]. Arctic regions are predicted to become thermally suitable through 2100 under CO_2_ emission scenario projections [26]. In contrast, regions south of the Brittany coast of France are predicted to become unsuitable [26], as temperatures will rise beyond the species’ potential for thermal acclimatization [41]. The susceptibility of *F. serratus* to climate change is expected to vary regionally, given the species’ regional patterns of genetic diversity [42], in combination with low gene flow between local populations [43].

Genetic diversity of *F. serratus* is highest in the two former, large glacial refugia (20–18 thousand years ago (kya)) in Southwest Ireland, and Brittany [42, 43]. The third refugium in the Northwest Iberian peninsula is characterized by a high proportion of private alleles, and currently represents the species’ isolated trailing edge, where recurrent cycles of thermally induced extinction and recolonization have eroded genetic diversity [42, 43]. Currently, sea surface temperatures reach 22 °C, and although below the lethal limit of *F. serratus* (25 °C) [40, 44], inhibit growth, physiological performance and reproductive capacity [45–48].

Genetic diversity of *F. serratus* decreases from its mid-range of distribution towards higher latitudes and is lowest in leading edge populations in northern Norway [42, 43]. Low genetic diversity in leading-edge populations is explained by the populations’ relatively young age and their derivation from small founder populations that carried only a subset of the genetic variation from glacial refugia to the north after the ice retreated, ca. 15–10 kya.

While *N*_*e*_ is a good indicator for temporal genetic stability, its estimation relies on temporally-spaced genetic data [49]. Due to this complication in sampling design, *N*_*e*_ of *F. serratus* has been estimated in only a single population close to Bergen (Norway) over eight years [39]. The estimated *N*_*e*_ between 73 and 386 was regarded insufficient for long-term survival under environmental change [39]. However, a thorough appraisal of the spatial distribution and temporal stability of *N*_*e*_ and genetic diversity throughout the species’ latitudinal range of distribution cannot be inferred from a single location.

Estimating climate change susceptibility in a species with low gamete/zygote dispersal requires to assess temporal genetic stability across its latitudinal and thermal range of distribution. In this study, we estimated *N*_*e*_ of *F. serratus* across latitude and temperature at seven locations with decadal sampling (2000 and 2010), a period in which Europe experienced three heat waves in 2003, 2006, and 2010 [50–52]. Here we evaluate whether range shifts in the north or strong selection pressures in the south have resulted in measurable changes in genetic diversity and population structure. In populations that are dominated by genetic drift and with small adaptive potential, we expected to find a decline in genetic diversity over the past decade. Finally, we discuss whether genetic diversity may be sufficiently stable to buffer environmental change and mitigate the current range shift predictions.

## Methods

### Sampling

**Fig. 1:**
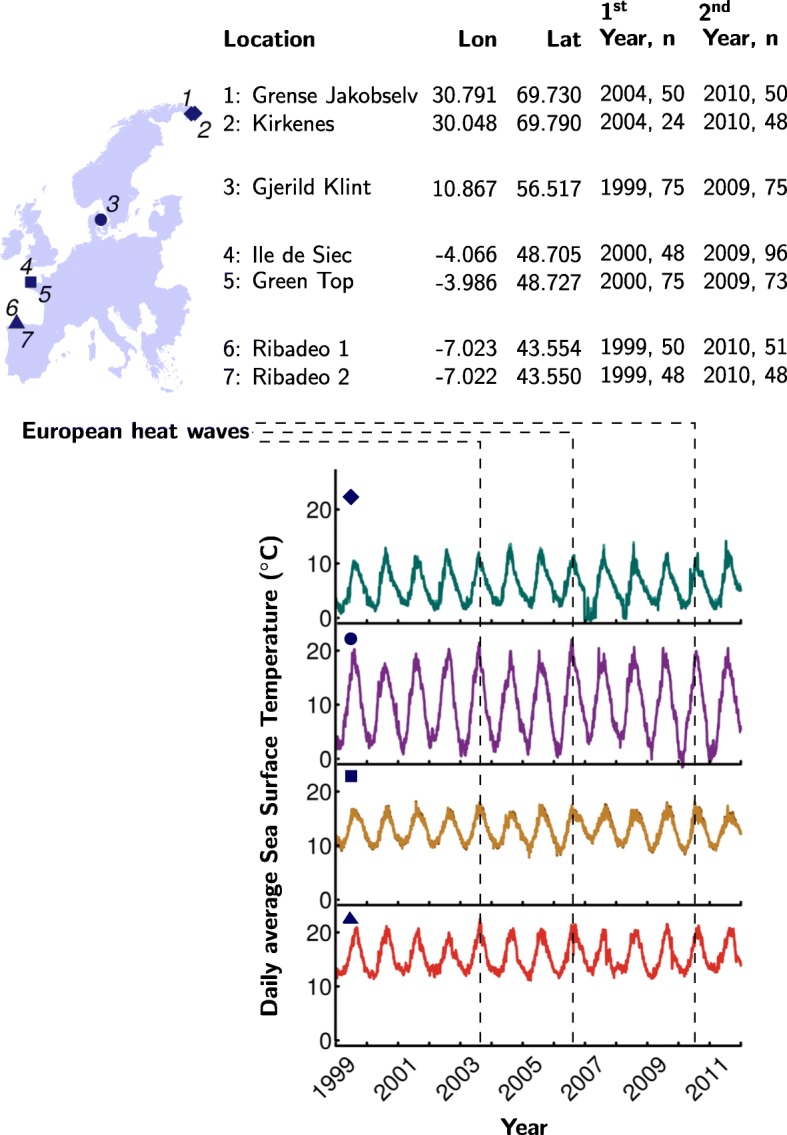
Sampling sites. Coordinates, years of collection, sampling sizes (n), and daily average sea surface temperatures (SST) at each of the seven sampling sites. SSTs were identical between the two Norwegian sampling sites as well as between the two French and the two Spanish sampling sites. Summer temperatures were exceptionally high at the Danish and Spanish sampling sites during the first two of three heat waves that Europe experienced in years 2003, 2006, and 2010

Individuals were sampled ca. ten years apart from the same seven populations spanning the latitudinal distribution of *F. serratus* (Fig. 1). Ethical approval is not required for research work with the seaweed/macroalga *F. serratus*. Field collections did not require specific permits and the species is neither endangered nor protected. Sampling involved removing a thumbnail-sized piece of tissue from ca. 50 to 100 individuals at each sampling site and did not threaten either the individual or the local population. Live samples were never transferred to other countries or locations within any of the countries. In all cases the specimens were collected within the context of various grants (see funding information) that involved at least one of the co-authors and one or more colleagues from the country where the collection was made.

Variability in daily average sea surface temperatures and surface air temperatures at the sampling locations (Fig. 1, Additional file 1), recorded from 1999 to 2011, were extracted from the NOAA/OI/SST/V2 dataset (0.25° resolution, described in [53]) and the CPC Global Temperature dataset (0.5° resolution) provided by NOAA/OAR/ESRL/PSD, Boulder, Colorado, USA, [54]). Thermal variability was replicated in the two Norwegian, the two French, and the two Spanish samples, respectively. In Denmark, only a single population was sampled at two time points. Individual tissues were blotted dry and stored in silica prior to transport for subsequent DNA extraction.

### Microsatellite genotyping

DNA was extracted from 2 mg silica dried tissue according to [55] with the modifications described in [56], followed by a purification step with the OneStep-96 PCR Inhibitor Removal Kit (Zymo Research, Irvine, USA) and a 1:3 dilution of the purified product. The samples were genotyped for a total of 31 microsatellite markers: 11 anonymous loci (L20, L38, L58, and L94 described in [57]; B113, B128, E6, E9, D39, A198, and F4 described in [58]) and 20 loci linked to expressed sequence tags (ESTs: F12, F22, F34, F36, F60, F45, F50, F17, F72, F49, F14, F21, F58, F19, F37, F65, F59, F69, F9, and described in [56]) (Additional file 2).

Polymerase chain reactions (PCRs) with 5 μl total volume contained 1 μl purified DNA template, 1.34 μl nuclease-free Water (Ambion, Thermo Fisher Scientific), 2.5 μl of AmpliTaq Gold 360 MM (Applied Biosystems, Life Technologies) and 0.08 μl of each forward and reverse primer (each primer at 20 μM; forward primer labeled with 6FAM, NED, PET or VIC; Applied Biosystems, Life Technologies). PCR was performed in a Veriti 96-Well Thermal Cycler (Applied Biosystems, Life Technologies). The conditions are depicted in Additional file 3 and specified for each marker in Additional file 2.

The fragment lengths were determined on an ABI 3500xl Genetic Analyzer from 1 μl of diluted PCR products (specified for each marker in Additional file 2) mixed with 8.9 μl of HiDi Formamide (Life Technologies) and 0.1 μl of Gene Scan 500 LIZ Size Standard (Life Technologies) after 5 min denaturation at 95 °C. Allele calling was performed with the GeneMapper v 4.1 Software (Applied Biosystems, Thermo Fisher Scientific).

### Data analysis

The microsatellite raw dataset (Additional file 4) was corrected for allelic dropout with a Maximum Likelihood approach [59] using the program MicroDrop [60]. From the corrected data (Additional file 5), nine markers E9, F14, F17, F36, F37, F59, F60, F65, and L20 were removed from the full set of 31 markers before further analyses with the remaining 22 markers because the proportion of missing data for the excluded markers exceeded 12% in at least one of the populations.

#### Diversity estimates

Average locus heterozygosity *H*_*exp*_ (bias-corrected [61]), allelic richness *α* (the average number of alleles per locus) and multi-locus heterozygosity (*MLH*), the number of heterozygous loci per individual divided by the number of loci, were calculated for each sampling location. Regional estimates were obtained after pooling the two spatial samples from each of the Norwegian, Spanish and French regions. Regional estimates were not possible for the Danish region because only one population was sampled. *H*_*exp*_ was calculated with the R package ‘DEMEtics’ [62], and *α* was estimated with the R package ‘PopGenReport’. For local estimates, *α* was normalized to a sample size of 24, the smallest number samples in a population. For regional estimates *α* was normalized to a sample size of 24, and additionally, to a sample size of 50. *MLH* was estimated with the R package ‘InbreedR’. Inbreeding coefficients *F*_*IS*_ [63] were estimated with the R package ‘Demerelate’ and tests for significant deviation from 0 were based on 1000 iterations. We tested for significant temporal changes of *H*_*exp*_, *α*, *F*_*IS*_, and *MLH* at each sampling location with Wilcoxon rank sum tests in R [64]. To assess temporal evolution of diversity estimates, we tested for correlation between current and historical local measures with a Spearman’s rank correlation in R [64]. Additionally, we tested for significant differences between average present-day and historical values using Wilcoxon Rank Sum tests in R [64].

Effective population sizes (*N*_*e*_) were estimated with an assumed generation time of 2 years [65] with the R package ‘NB’ after removing loci with only one allele: Locus F9 for the Kirkenes population, locus F72 for the Ribadeo1 population and loci F21 and F72 for the Ribadeo2 population.

#### Genetic differentiation

Population structure was determined with Bayesian clustering methods implemented in the software Structure v2.3.4 [66]. Acceptance of six clusters (K) was determined with the *δ* K Method [67] in the R package ‘pophelper’ [68].

Temporal genetic changes at each sampling location and geographic genetic differentiation within and between all historical and recent samples were estimated by the fixation index *F*_*ST*_ [69] using GENETIX 4.05 [70] and the differentiation index *D*_*est*_ [71]) using the R package ‘DEMEtics’ 0.8–7 [62]. *D*_*est*_ more correctly measures the true genetic differentiation compared with *F*_*ST*_ for multi-allelic markers such as microsatellites [62, 71]. Statistical significance of the pairwise comparisons was based on 10,000 permutations for *F*_*ST*_ and on 1000 Bootstrap repeats for *D*_*est*_. To assess temporal stability of geographic differentiation, we tested for correlation between recent and historical *F*_*ST*_ and *D*_*est*_ values with Spearmans’s rank correlation in R [64]. Additionally, we tested for significant differences between average present-day and historical values using Wilcoxon Rank Sum tests in R [64]. Finally, we tested for correlation between temporal genetic differentiation *(F*_*ST*_, *D*_*est*_*)* and *N*_*e*_ with Pearson’s product moment correlation in R [64].

## Results

### Genetic structure

**Fig. 2:**
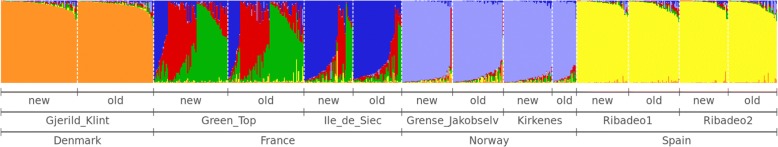
Clustering of samples. Sample assignment to six clusters (colors) with the program Structure shows consistent geographic differentiation between sampling times. Here, new and old refers to the two sampling years specified in Fig. 1

**Fig. 3:**
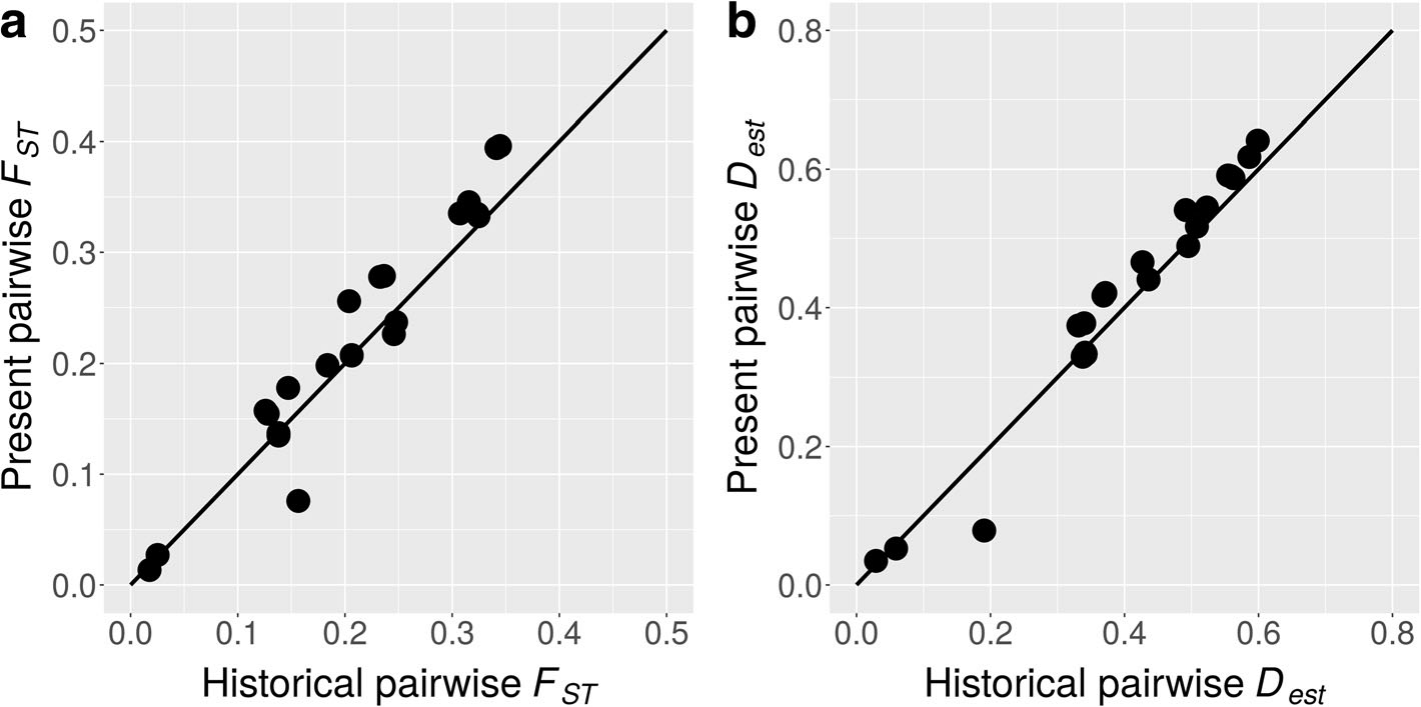
Present and historical genetic differentiation. Population differentiation estimated by *F*_*ST*_ (**a**) and *D*_*est*_ (**b**) with a 1:1 reference line

Bayesian clustering with the program Structure revealed clear differences between regions but not with time (Fig. 2). Historical and present-day *F*_*ST*_ values were strongly positively correlated (*r* = 0.93, *p* < 0.00001), and the overall historical *F*_*ST*_ value (0.21) did not differ significantly (*p* = 0.567) from the present-day value (0.22), indicating that spatial genetic differentiation between populations was globally consistent over time (Fig. 3a). Historical and present-day *D*_*est*_ values supported these findings as the overall values (0.40 and 0.42, respectively) did not differ significantly (*p* = 0.636) and were positively correlated (*r* = 0.97, *p* < 0.00001, Fig. 3b). Isolation by distance was indicated by stronger differentiation among than within countries (Additional file 6).

Temporal changes, however, were noted on a local level. Local differentiation between the Norwegian populations decreased from 2004 to 2010 (Additional file 6). All but the French population ‘Ile de Siec’ changed significantly in genetic variation over time, as indicated by significant changes in *F*_*ST*_ and *D*_*est*_ (Additional file 7). The Spanish population ‘Ribadeo2’ showed significant temporal change in *F*_*ST*_ but not in *D*_*est*_.

### Genetic variation/diversity

**Fig. 4:**
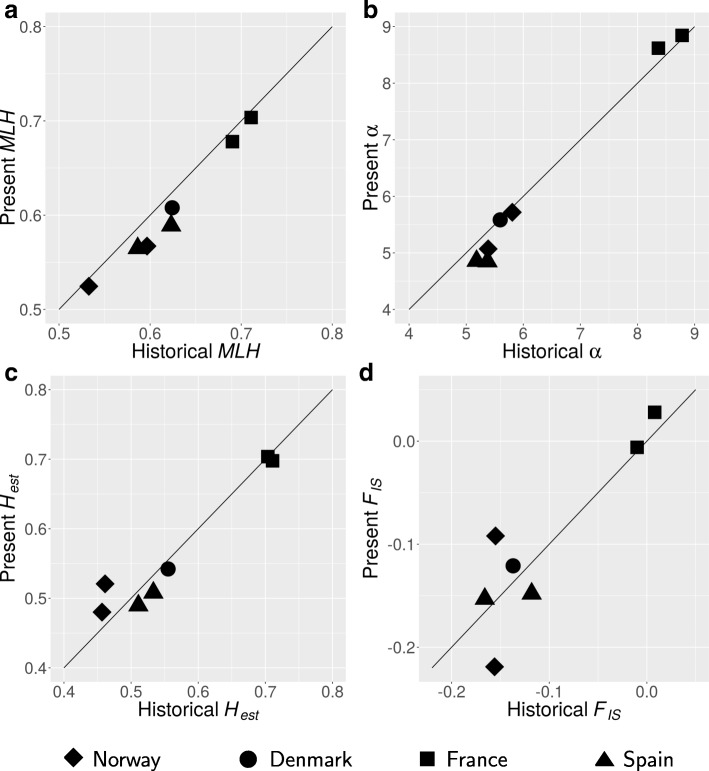
Genetic diversity across latitudes. Present and historical diversity estimates of **a**) multi-locus heterozygosity (*MLH*), **b**) allelic richness (*α*), **c**) expected heterozygosity (*H*_*exp*_), and **d**) inbreeding (*F*_*IS*_), with 1:1 reference lines representing unchanged temporal evolution

Stable population diversities through time were indicated by significant correlations of historical and present-day intra-population diversity indices (Fig. 4; *H*_*exp*_: *r* = 0.86*, p* = 0.02, *MLH: r* = 1, *p* = 0.0004; *α: r* = 0.96, *p =* 0.003; *F*_*IS*_: *r* = 0.82, *p* = 0.03). Moreover, average present-day values did not differ significantly (*p* > 0.05) from average historical values (*H*_*exp*_: present = 0.56, historical = 0.56; *MLH*: present = 0.61, historical = 0.62; *α*: present = 6.22, historical = 6.36; *F*_*IS*_: present = − 0.10, historical = − 0.10).

Local and regional diversity estimates (Additional file 8) were highest in France and lower at the northern and southern distribution edges (Fig. 4). Regional *α* estimates (standardized to 50 samples) exceeded local estimates (standardized to 24 samples) in all regions (Additional file 8).

**Fig. 5:**
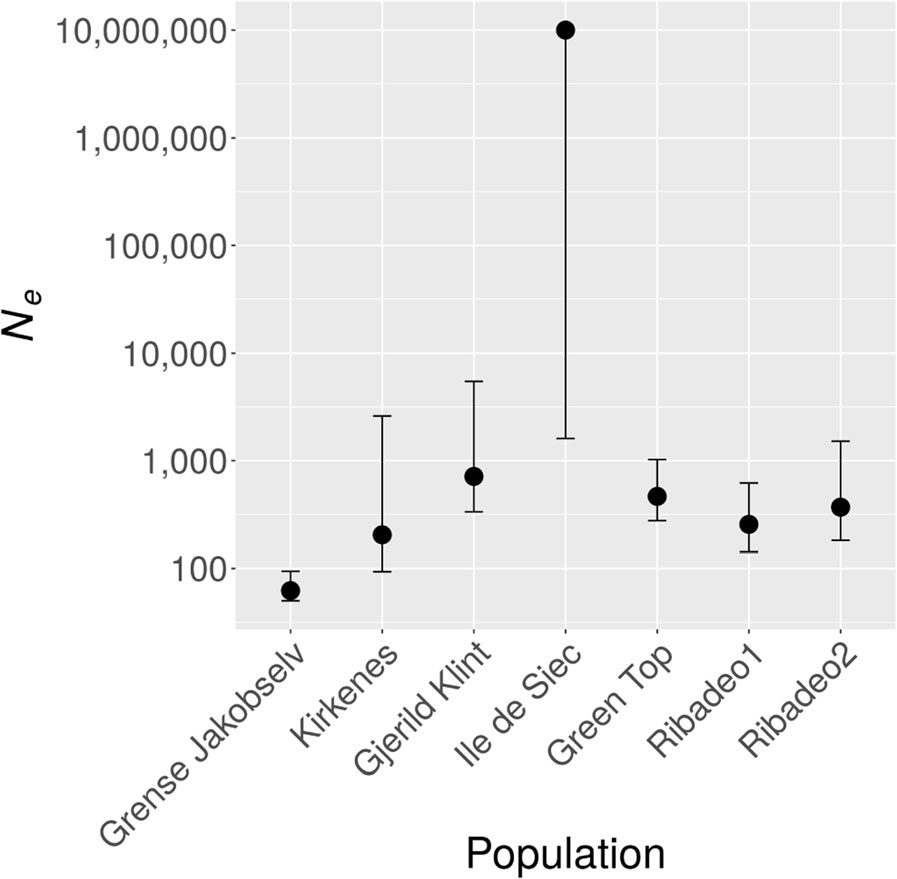
Effective population sizes across latitudes. Effective population size (*N*_*e*_) at each sampling location with 95% confidence intervals

Effective population size (*N*_*e*_) was highest in the French population ‘Ile de Siec’ (*N*_*e*_ *= 10,000,000*) and lowest in the Norwegian population ‘Grense Jakobselv’ (*N*_*e*_ *= 62*) (Fig. 5, Additional file 7). *N*_*e*_ for the other populations ranged from 700 to 200 in the order: Gjerild Klint > Green Top > Ribadeo2 > Ribadeo1 > Kirkenes. At both sampling time points, none of the diversity estimates were significantly correlated with effective population size (all *p* > 0.09). The temporal decrease in *MLH* in Ribadeo2 was strong but not significant (*p* = 0.051, Additional file 7). The *F*_*IS*_ in ‘Kirkenes’ was significantly negative (*p* = 0.043, Additional file 8).

## Discussion

The spatial distribution of genetic diversity has important implications for conservation and management as it reveals a species’ strong and weak points with regards to stability and evolutionary capacity [72–74]. Given the key ecological role of *F. serratus* [30–32, 75–77], the positive effect of genetic diversity may reach beyond the species level to affect community structure and increase species richness and stability of the entire associated ecosystem [78–81]. We are not aware of seaweed studies that have investigated positive ecosystem effects of genetic diversity, but genetic diversity enhanced heat-stress survival in germlings of *Fucus vesiculosus* [27]. Furthermore, in the habitat forming seagrass *Z. marina*, genotypic diversity not only enhanced biomass production, but also abundance of the associated fauna under near-lethal water temperatures [82] and community resistance to grazing [83]. Thus, maintaining genetic diversity in *F. serratus* is also expected to be important for conservation and management of the entire intertidal ecosystem along temperate rocky shores. Across the latitudinal range of *F. serratus*, genetic diversity and differentiation remained stable for 5–10 generations at regional scales, and in all but the Norwegian region at local spatial scales (Figs. 2, 3, 4). This suggests that, despite low gene flow between populations, effective population sizes have remained large enough to maintain genetic variation at least on the short term. Temporal genetic differentiation was systematically lower than local differentiation, and 1–2 orders of magnitude lower than regional differentiation (Additional file 9). This implies that temporal snapshots provide valuable information for conservation management of fucoid seaweeds, as they reliably reflect diversity and differentiation patterns for at least a decade.

### *N*_*e*_ comparisons

In all but the Norwegian populations, *N*_*e*_ was estimated as > 260, a size reported as the median estimate for stable populations in over 83 studies spanning a diverse range of taxa [36]. This suggests low sensitivity to genetic stochasticity [36] in all but the northern edge populations of *F. serratus*. As in most studies, the precision of *N*_*e*_ decreased as *N*_*e*_ increased (Fig. 5) [36, 84, 85]. Local differentiation in *F. serratus* is one of the most important assumptions of the employed ‘temporal’ method to estimate *N*_*e*_, in which neutral genetic change over time is expected to be inversely proportional to *N*_*e*_. Discrete generations are another important assumption of the ‘temporal’ method. Overlapping generations are unlikely to cause a significant downward bias of *N*_*e*_ when more than 4 generations lie between the temporal samples [49]. This can be expected for most of our temporal samples, assuming a generation time of 1–2 years [65, 86] and a time span of 6–11 years between sampling. Thus, our sample-size-corrected estimates can be regarded as unbiased and indicative of a ‘real’ decline in *N*_*e*_ from the species’ mid-range of distribution to its range-edges.

An *N*_*e*_ > 1000, as in the French ‘Ile de Siec’ population, is large enough to ensure evolutionary potential in perpetuity [87], and is likely to provide the best source for adaptive genetic rescue of threatened and declining populations [38, 88]. However, large *N*_*e*_ estimates are commonly associated with a high uncertainty [36, 85]. Accordingly, the point estimate of *N*_*e*_ in the ‘Ile de Siec’ population (ca. 10 Million) has a wide confidence interval as compared with the other populations (Fig. 5, Additional file 7). Consequently, the point estimate is unlikely to be the true value in this population, but is certainly > 1000, and higher than in any other measured populations. The reason for this outlier value is not due to high diversity, since this is comparable to the other French population (Fig. 4a-d), but the high stability in allele frequencies over time. Indeed, the ‘Ile de Siec’ population was the only population for which temporal genetic differentiation was non-significant (Additional file 7).

*N*_*e*_ in the other mid-range populations, > 500, may be sufficient in the mid-term [36, 87, 89] to mitigate the predicted extinction by the end of the twenty-first century [26]. However, given that summer temperatures are predicted to rise above the thermal tolerance limit of *F. serratus* in Brittany within the next 200 years [26, 41], it is important to track its fitness in this region in order to implement early conservation measures in case it loses its current stability.

An *N*_*e*_ of 50–100 was regarded necessary for a population to minimize inbreeding depression and associated problems such as accumulation of deleterious mutations and loss of variation [36, 87]. However, despite *N*_*e*_ *< 60* in the Norwegian ‘Grense Jakobselv’ population, genetic diversity remained stable for both Norwegian populations over six years and neither population was inbred. In contrast, a previous study on a southern Norwegian population reported significant loss of *N*_*e*_ from 2000 to 2008 and concluded that an *N*_*e*_ between 73 and 386 was insufficient for long-term survival under environmental change [39]. Stable diversity despite small *N*_*e*_ in our two northern Norwegian populations may be ascribed to regional gene flow, suggested by a reduction in genetic differentiation between the two Norwegian populations from 2004 to 2010 and significant outbreeding (negative *F*_*IS*_) in the ‘Kirkenes’ population in 2010. Thus, regional gene flow may uncouple *N*_*e*_ from genetic stochasticity effects at the species’ Northern edge of distribution.

### Diversity comparisons

As expected for neutral loci, genetic diversity was positively related with *N*_*e*_ [36, 38]. Both regional and local diversities are highest in Brittany and make the range-center of *F. serratus* less sensitive to genetic drift [36, 37]. A decline in genetic diversity towards the northern and southern range-edges is in accordance with the species’ biogeographic history [42].

Low genetic diversity does not necessarily lower the evolutionary potential of *F. serratus* to adapt to Arctic shores [26]. The evolutionary potential, survival, and expansion rate of low-diversity leading edge populations [3] may decrease when deleterious mutations accumulate at expansion range fronts and create a so-called ‘expansion load’ [7, 9]. On the other hand, survival may well be enhanced by the ‘surfing’ of favorable mutations that can rapidly increase in frequency over iterated founder events [4, 5, 90]. An additional consideration is that source populations of Arctic colonists may not be located at the species’ northern edge, but within European harbors with frequent shipping, fishing, and cruise boat traffic to and from the northern polar regions.

Our results have clear implications for the conservation of *F. serratus* at its southern edge. Reductions in *MLH* from 1999 to 2010 were close to significant (*p* = 0.0051/0.134 for Ribadeo2/Ribadeo1, respectively), although, *H*_*exp*_, *α*, and *F*_*IS*_ remained temporally stable. This agrees with stability of *H*_*exp*_ and *α* over 7–9 years in fragmented southern edge populations of the kelp species *Laminaria digitata* [91], and is likely due to the measures’ insensitivity to the effects of population bottlenecks [92]. In other words, while the polymorphic state of loci and the diversity of alleles did not decline, alleles occurred more frequently in a homozygous state in the recent samples. In theory, the decline in *MLH* might be explained by increased selection pressure for heat-tolerance, although there is only indirect experimental evidence for this. Acclimation potential to further thermal stress is likely impeded in this population by chronically high expression of heat shock protein genes [22, 23, 41, 93]. Between 2000 and 2010, the Ribadeo1 population experienced a 90% decline in abundance [26]. Although stable local differentiation favors ecotypic differentiation in thermal stress tolerance [41], heat-stress is becoming too extreme at the southern edge.

The value of conserving the southern edge of *F. serratus* may be high [94]. Because of its separation from Brittany by the uninhabitable sandy warm shores of the Bay of Biscay, the Northwest Iberian glacial refugium did not contribute to postglacial recolonizations of ice-free northern shores, and, thus, preserves unique genetic variation [42]. The conservation value of the species’ southern edge becomes even more apparent when considering that small-scale population structure increases the species’ regional diversity above local diversity within single populations (Additional file 8). High regional diversity, despite low within-population diversity, was previously reported for the southern distribution edge of the seagrass *Zostera marina* [94, 95]. We are not aware of studies that explicitly addressed this effect in macroalgae, although increased local differentiation at the southern edge of the kelp *Laminaria digitata* [91] can be expected to increase regional variation as well. Thus, with the loss of its southern edge, the species’ can be expected to lose its most heat-adapted populations sustaining unique genetic variation.

## Conclusions

Temporal snapshots of genetic diversity and structure in *F. serratus* populations spanning its latitudinal range reliably reflect patterns across local and regional spatial scales and across various thermal backgrounds for at least one decade. Stable small-scale structure enhances regional genetic diversity throughout the species’ range of distribution and is a potential driver for local adaptation [36] that may explain species radiation and diversity in the genus Fucus [96–98].

*MLH* appears to be the most stress-sensitive measure of diversity, displaying a strong decline at the species’ southern edge of distribution. As sandy warm shores separate the Iberian southern edge from the genetically diverse Brittany region, genetic rescue by the influx of genetic variation [38, 88] might only be possible if initiated by conservation efforts.

Increasing temperatures are likely the major cause for the decline not only of *F. serratus,* but also other intertidal and subtidal macroalgae in Northwest Iberia [28, 99–101], as well as temperate seaweeds worldwide [102]. Kelp species may maintain genetic diversity to a certain degree in southern edges by escaping to deep-water refugia to avoid rising temperatures in shallow waters [103]. Accordingly, in Northern Portugal, increasing air-temperature stress depresses the upper boundary limit of *F. serratus* [104]. However, intertidal seaweeds are less adapted to low light conditions and, thus, have low potential to escape into deeper waters. Another factor that impedes survival of southern edge populations in fucoid seaweeds is their reproductive strategy with fewer gametes and lower dispersal (< 12 m from parental sites [45, 46]) as compared with kelps that release billions of spores dispersing several kilometers [105, 106]. We suspect that without the influx of genetic variation from Brittany, intertidal habitat-forming macroalgae, such as *F. serratus*, may largely disappear from southern edges but retain potential to persist in small subtidal bottleneck populations in cool upwelling regions [107].

## Additional files

Additional file 1: Surface air temperatures. Daily average surface air temperatures (SAT) at each of the seven sampling sites from 1999 to 2011 with gaps in year 2006 for the French and Spanish sampling sites. SATs were identical between the two Norwegian sampling sites as well as between the two French and the two Spanish sampling sites. (PDF 513 kb)

Additional file 2: Microsatellite markers. Characteristics of each microsatellite marker, including cycling conditions and multiplexing. (XLSX 8 kb)

Additional file 3: PCR cycling protocols. Time-release (a) and no-time-release (b) PCR cycling protocols. In the time-release protocol, the heat-activated DNA-polymerase was progressively released during the thermal cycling process. Annealing temperatures and number of cycles indicated with an X are specified for each marker in Additional file 2. (PDF 34 kb)

Additional file 4: Microsatellite raw data. Microsatellite genotypes in Structure format. The first row contains the names of all 31 markers. The following rows contain the individual genotype data. Each individual is represented in 2 consecutive rows. The first column contains the name of the individual, the second row contains the population number that individual belong to. The following 31 columns show the alleles of each marker as microsatellites base pair lengths. The population numbers (1–14) refer to the following sampling locations and times: 1) Gjerild Klint, present-day; 2) Gjerild Klint, historical; 3) Green Top, present-day; 4) Green Top, historical; 5) Ile de Siec, present-day; 6) Ile de Siec, historical; 7) Grense Jakobselv, present-day; 8) Grense Jakobselv, historical; 9) Kirkenes, present-day; 10) Kirkenes, historical; 11) Ribadeo 1, present-day; 12) Ribadeo 1, historical; 13) Ribadeo 2, present-day; 14) Ribadeo 2, historical. (TXT 195 kb)

Additional file 5 Corrected microsatellite data. Microsatellite data corrected for allelic dropout in Structure format. The first row contains the names of all 31 markers. The following rows contain the individual genotype data. Each individual is represented in 2 consecutive rows. The first column contains the name of the individual, the second row contains the population number that individual belong to. The following 31 columns show the alleles of each marker as microsatellites base pair lengths. The population numbers (1–14) refer to the same sampling locations and times as in Additional file 4. (TXT 198 kb)

Additional file 6: Spatial differentiation. Regional and local genetic differentiation between sampling sites in historical and present samples estimated by *F*_*ST*_ and *D*_*est*_ with *p* values. (XLSX 10 kb)

Additional file 7: Temporal changes. Estimates of effective population size (*N*_e_), temporal genetic differentiation (*F*_*ST*_ and *D*_*est*_), and *p* values for temporal changes in diversity measures at each sampling site. (XLSX 6 kb)

Additional file 8: Diversity estimates. Diversity estimates, including heterozygosity (*H*_exp_), allelic richness (*α*), and multi-locus heterozygosity (*MLH*), for each location and region with standard errors, and inbreeding coefficients *F*_*IS*_ with *p* values for each population. (XLSX 8 kb)

Additional file 9: Temporal versus local genetic differentiation. Temporal genetic change in comparison to local and regional genetic differentiation(*F*_*ST*_ and *D*_*est*_) for each population and sampling time point (historical and present). (XLSX 6 kb)

## Abbreviations

*CI*: Confidence interval
*D*_*est*_: Differentiation index measuring genetic differentiation
DNA: Deoxyribonucleic acid
EST: Expressed sequence tag
*F*_*IS*_: Inbreeding coefficient
*F*_*ST*_: Fixation index measuring genetic differentiation
*H*_*exp*_: Expected heterozygosity
kya: thousand years ago
*MLH*: Multi locus heterozygosity
*N*_*e*_: Effective population size
PCR: Polymerase chain reaction
SAT: Surface air temperature
SST: Sea surface temperature
α: Allelic richness

## References

[1] Orr HA, Unckless RL (2008). Population extinction and the genetics of adaptation. Am Nat JSTOR.

[2] Teotónio H, Chelo IM, Bradić M, Rose MR, Long AD (2009). Experimental evolution reveals natural selection on standing genetic variation. Nat Genet.

[3] Pauls SU, Nowak C, Bálint M, Pfenninger M (2013). The impact of global climate change on genetic diversity within populations and species. Mol Ecol.

[4] Excoffier L, Ray N (2008). Surfing during population expansions promotes genetic revolutions and structuration. Trends Ecol Evol.

[5] Klopfstein S, Currat M, Excoffier L (2006). The fate of mutations surfing on the wave of a range expansion. Mol Biol Evol.

[6] Travis JMJ, Münkemüller T, Burton OJ, Best A, Dytham C, Johst K (2007). Deleterious mutations can surf to high densities on the wave front of an expanding population. Mol Biol Evol.

[7] Peischl S, Dupanloup I, Kirkpatrick M, Excoffier L (2013). On the accumulation of deleterious mutations during range expansions. Mol Ecol.

[8] Bosshard L, Dupanloup I, Tenaillon O, Bruggmann R, Ackermann M, Peischl S (2017). Accumulation of deleterious mutations during bacterial range expansions. Genetics.

[9] Gilbert KJ, Sharp NP, Angert AL, Conte GL, Draghi JA, Guillaume F (2017). Local adaptation interacts with expansion load during range expansion: maladaptation reduces expansion load. Am Nat.

[10] Walter RP, Aykanat T, Kelly DW, Shrimpton JM, Heath DD (2009). Gene flow increases temporal stability of Chinook salmon (*Oncorhynchus tshawytscha*) populations in the upper Fraser River, British Columbia, Canada. Can J Fish Aquat Sci.

[11] Larsson LC, Laikre L, André C, Dahlgren TG, Ryman N (2010). Temporally stable genetic structure of heavily exploited Atlantic herring (*Clupea harengus*) in Swedish waters. Heredity (Edinb).

[12] Jónsdóttir ÓDB, Daníelsdóttir AK, Nævdal G (2001). Genetic differentiation among Atlantic cod (*Gadus morhua* L.) in Icelandic waters: temporal stability. ICES J Mar Sci.

[13] Ruzzante DE, Taggart CT, Doyle RW, Cook D (2001). Stability in the historical pattern of genetic structure of Newfoundland cod (*Gadus morhua*) despite the catastrophic decline in population size from 1964 to 1994. Conserv Genet.

[14] Waples RS (1990). Conservation genetics of Pacific Salmon. II. Effective population size and the rate of loss of genetic variability. J Hered.

[15] Piertney SB, Carvalho GR (1995). Microgeographic genetic differentiation in the intertidal isopod *Jaera albifrons* leach. II. Temporal variation in allele frequencies. J Exp Mar Bio Ecol.

[16] Toonen RJ, Grosberg RK. Causes of chaos: spatial and temporal genetic heterogeneity in the intertidal anomuran crab Petrolisthes cinctipes. In: Crustacean Issues: Phylogeography and Population Genetics in Crustacea (eds Koenemann S, Held C, Schubart C). Boca Raton: CRC Press; 2011:75–107.

[17] Lee HJ, Boulding EG (2007). Mitochondrial DNA variation in space and time in the northeastern Pacific gastropod, *Littorina keenae*. Mol Ecol.

[18] Barcia AR, López GE, Hernández D, García-Machado E (2005). Temporal variation of the population structure and genetic diversity of *Farfantepenaeus notialis* assessed by allozyme loci. Mol Ecol.

[19] Reynolds LK, Stachowicz JJ, Hughes AR, Kamel SJ, Ort BS, Grosberg RK (2017). Temporal stability in patterns of genetic diversity and structure of a marine foundation species (*Zostera marina*). Heredity (Edinb). Nat Publ Group.

[20] Lessios H, Weinberg J, Starczak V (1994). Temporal variation in populations of the marine isopod Excirolana: how stable are gene frequencies and morphology?. Evolution (N Y).

[21] Helmuth B, Mieszkowska N, Moore P, Hawkins SJ (2006). Living on the edge of two changing worlds: forecasting the responses of rocky intertidal ecosystems to climate change. Annu Rev Ecol Evol Syst.

[22] Tomanek L (2010). Variation in the heat shock response and its implication for predicting the effect of global climate change on species’ biogeographical distribution ranges and metabolic costs. J Exp Biol.

[23] Somero GN (2010). The physiology of climate change: how potentials for acclimatization and genetic adaptation will determine “winners” and “losers.”. J Exp Biol.

[24] Helmuth B, Broitman BR, Blanchette CA, Gilman S, Halpin P, Harley CDG (2006). Mosaic patterns of thermal stress in the rocky intertidal zone: implications for climate change. Ecol Monogr Eco Soc America.

[25] Somero GN (2005). Linking biogeography to physiology: evolutionary and acclimatory adjustments of thermal limits. Front Zool.

[26] Jueterbock A, Tyberghein L, Verbruggen H, Coyer JA, Olsen JL, Hoarau G (2013). Climate change impact on seaweed meadow distribution in the North Atlantic rocky intertidal. Ecol Evol.

[27] Al-Janabi B, Kruse I, Graiff A, Karsten U, Wahl M (2016). Genotypic variation influences tolerance to warming and acidification of early life-stage *Fucus vesiculosus* L. (Phaeophyceae) in a seasonally fluctuating environment. Mar Biol.

[28] Assis J, Lucas AV, Bárbara I, Serrão EÁ (2016). Future climate change is predicted to shift long-term persistence zones in the cold-temperate kelp *Laminaria hyperborea*. Mar Environ Res.

[29] Jueterbock A, Smolina I, Coyer JA, Hoarau G (2016). The fate of the Arctic seaweed *Fucus distichus* under climate change: an ecological niche modeling approach. Ecol Evol..

[30] Christie H, Norderhaug KM, Fredriksen S (2009). Macrophytes as habitat for fauna. Mar Ecol Prog Ser.

[31] Fredriksen S, Christie H, Sæthre BA (2005). Species richness in macroalgae and macrofauna assemblages on *Fucus serratus* L. (Phaeophyceae) and *Zostera marina* L. (Angiospermae) in Skagerrak, Norway. Mar Biol Res.

[32] Harley CDG, Anderson KM, Demes KW, Jorve JP, Kordas RL, Coyle TA (2012). Effects of climate change on global seaweed communities. J Phycol.

[33] Wahl M, Jormalainen V, Eriksson BK, Coyer JA, Molis M, Schubert H, Lesser M (2011). Stress ecology in *Fucus*: abiotic, biotic and genetic interactions. Adv Mar Biol.

[34] Wright S (1990). Evolution in mendelian populations. Bull Math Biol.

[35] Crow JF, Kimura M. An introduction to population genetics theory. Cours l’University Oslo Dep Informatics. 1970;591 10.2307/1529706.

[36] Palstra FP, Ruzzante DE (2008). Genetic estimates of contemporary effective population size: what can they tell us about the importance of genetic stochasticity for wild population persistence?. Mol Ecol.

[37] Charlesworth B (2009). Fundamental concepts in genetics: effective population size and patterns of molecular evolution and variation. Nat Rev Genet.

[38] Bijlsma R, Loeschcke V (2012). Genetic erosion impedes adaptive responses to stressful environments. Evol Appl.

[39] Coyer JA, Hoarau G, Sjotun K, Olsen JL (2008). Being abundant is not enough: a decrease in effective population size over eight generations in a Norwegian population of the seaweed, *Fucus serratus*. Biol Lett.

[40] Lüning K, Yarish C, Kirkman H (1990). Seaweeds: their environment, biogeography, and ecophysiology. Wiley-Interscience, New York, USA.

[41] Jueterbock A, Kollias S, Smolina I, Fernandes JMO, Coyer JA, Olsen JL (2014). Thermal stress resistance of the brown alga *Fucus serratus* along the North-Atlantic coast: Acclimatization potential to climate change. Mar Genomics.

[42] Hoarau G, Coyer JA, Veldsink JH, Stam WT, Olsen JL (2007). Glacial refugia and recolonization pathways in the brown seaweed *Fucus serratus*. Mol Ecol.

[43] Coyer JA, Peters AF, Stam WT, Olsen JL (2003). Post-ice age recolonization and differentiation of *Fucus serratus* L. (Phaeophyceae; Fucaceae) populations in Northern Europe. Mol Ecol.

[44] Lüning K (1984). Temperature tolerance and biogeography of seaweeds: the marine algal flora of Helgoland (North Sea) as an example. Helgoländer Meeresuntersuchungen.

[45] Arrontes J (1993). Nature of the distributional boundary of *Fucus serratus* on the north shore of Spain. Mar Ecol Prog Ser.

[46] Arrontes J (2002). Mechanisms of range expansion in the intertidal brown alga *Fucus serratus* in northern Spain. Mar Biol.

[47] Viejo RM, Martínez B, Arrontes J, Astudillo C, Hernández L (2011). Reproductive patterns in central and marginal populations of a large brown seaweed: drastic changes at the southern range limit. Ecography (Cop).

[48] Martínez B, Arenas F, Rubal M, Burgués S, Esteban R, García-Plazaola I (2012). Physical factors driving intertidal macroalgae distribution: physiological stress of a dominant fucoid at its southern limit. Oecologia.

[49] Waples RS, Yokota M (2007). Temporal estimates of effective population size in species with overlapping generations. Genetics.

[50] Schär C, Jendritzky G (2004). Hot news from summer 2003. Nature.

[51] Rebetez M, Dupont O, Giroud M (2009). An analysis of the July 2006 heatwave extent in Europe compared to the record year of 2003. Theor Appl Climatol.

[52] Barriopedro D, Fischer EM, Luterbacher J, Trigo RM, García-Herrera R (2011). The hot summer of 2010: redrawing the temperature record map of Europe. Science (80- ).

[53] Reynolds RW, Rayner NA, Smith TM, Stokes DC, Wang W (2002). An improved in situ and satellite SST analysis for climate. J Clim.

[54] Earth System Research Laboratory: Physical Sciences Division. https://www.esrl.noaa.gov/psd/. Accessed: 01 Jan 2013.

[55] Hoarau G, Coyer JA, Stam WT, Olsen JL (2007). A fast and inexpensive DNA extraction/purification protocol for brown macroalgae: technical article. Mol Ecol Notes.

[56] Coyer JA, Hoarau G, Beszteri B, Pearson G, Olsen JL (2009). Expressed sequence tag-derived polymorphic SSR markers for *Fucus serratus* and amplification in other species of *Fucus*. Mol Ecol Resour.

[57] Engel CR, Brawley SH, Edwards KJ, Serrão E (2003). Isolation and cross-species amplification of microsatellite loci from the fucoid seaweeds Fucus vesiculosus, F. serratus and Ascophyllum nodosum (Heterokontophyta, Fucaceae). Mol Ecol Notes.

[58] Coyer JA, Veldsink JH, Jones K, Stam WT, Olsen JL (2002). Characterization of microsatellite loci in the marine seaweeds, Fucus serratus and F. evanescens(Heterokontophyta, Fucaceae). Mol Ecol Notes.

[59] Wang C, Schroeder KB, Rosenberg NA (2012). A maximum-likelihood method to correct for allelic dropout in microsatellite data with no replicate genotypes. Genetics.

[60] Rosenberg lab at Standford University: MicroDrop program. http://rosenberglab.stanford.edu/microdrop.html. Accessed: 05 Jan 2017.

[61] Nei M (1978). Estimation of average heterozygosity and genetic distance from a small number of individuals. Genetics.

[62] Gerlach G, Jueterbock A, Kraemer P, Deppermann J, Harmand P (2010). Calculations of population differentiation based on G_ST_ and D: forget G_ST_ but not all of statistics. Mol Ecol.

[63] Wright S (1965). The interpretation of population structure by F-statistics with special regard to Systems of Mating. Evolution (N Y)..

[64] Core Team R (2017). R: A language and environment for statistical Computing R Found.

[65] Coyer JA, Hoarau G, Stam WT, Olsen JL (2007). Hybridization and introgression in a mixed population of the intertidal seaweeds *Fucus evanescens* and *F. serratus*. J Evol Biol.

[66] Falush D, Stephens M, Pritchard JK (2007). Inference of population structure using multilocus genotype data: dominant markers and null alleles. Mol Ecol.

[67] Evanno G, Regnaut S, Goudet J (2005). Detecting the number of clusters of individuals using the software STRUCTURE: a simulation study. Mol Ecol.

[68] Francis RM (2017). Pophelper: an R package and web app to analyse and visualize population structure. Mol Ecol Resour.

[69] Weir BS, Cockerham CC (1984). Estimating F-statistics for the analysis of population structure. Evolution (N Y).

[70] Belkhir K, Borsa P, Chikhi L, Raufaste N, Bonhomme F. GENETIX 4.05, logiciel sous Windows TM pour la génétique des populations. Lab. génome, Popul. Interact. CNRS Umr. Montpellier, France

[71] Jost L (2008). G_ST_ and its relatives do not measure differentiation. Mol Ecol.

[72] Lande R, Barrowclough GF, Soule ME (1987). Effective population size, genetic variation, and their use in population management. Viable Popul Conserv.

[73] Franklin IR, Frankham R (1998). How large must populations be to retain evolutionary potential?. Anim Conserv.

[74] Hare MP, Nunney L, Schwartz MK, Ruzzante DE, Burford M, Waples RS (2011). Understanding and estimating effective population size for practical application in marine species management. Conserv Biol.

[75] Chapman ARO (1995). Functional ecology of fucoid algae: twenty-three years of progress. Phycologia.

[76] Boaden PJS (1996). Habitat provision for meiofauna by *Fucus serratus* epifauna with particular data on the flatworm *Monocelis lineata*. Mar Ecol.

[77] Dijkstra JA, Boudreau J, Dionne M (2012). Species-specific mediation of temperature and community interactions by multiple foundation species. Oikos.

[78] Crutsinger GM (2006). Plant genotypic diversity predicts community structure and governs an ecosystem process. Science (80-).

[79] Hughes AR, Inouye BD, Johnson MTJ, Underwood N, Vellend M (2008). Ecological consequences of genetic diversity. Ecol Lett.

[80] Vellend M (2006). The consequences of genetic diversity in competitive communities. Ecology.

[81] Duffy JE, Godwin CM, Cardinale BJ (2017). Biodiversity effects in the wild are common and as strong as key drivers of productivity. Nature.

[82] Reusch TBH, Ehlers A, Hammerli A, Worm B (2005). Ecosystem recovery after climatic extremes enhanced by genotypic diversity. Proc Natl Acad Sci.

[83] Hughes AR, Stachowicz JJ (2004). Genetic diversity enhances the resistance of a seagrass ecosystem to disturbance. Proc Natl Acad Sci.

[84] Waples RS (1989). Temporal variation in allele frequencies: testing the right hypothesis. Evolution (N Y)..

[85] DeFaveri J, Merilä J (2015). Temporal stability of genetic variability and differentiation in the three-spined stickleback (*Gasterosteus aculeatus*). PLoS One.

[86] Coyer JA, Hoarau G, Skage M, Stam WT, Olsen JL (2006). Origin of *Fucus serratus* (Heterokontophyta; Fucaceae) populations in Iceland and the Faroes: a microsatellite-based assessment. Eur J Phycol.

[87] Frankham R, Bradshaw CJA, Brook BW (2014). Genetics in conservation management: revised recommendations for the 50/500 rules, red list criteria and population viability analyses. Biol Conserv.

[88] Bell G, Gonzalez A (2009). Evolutionary rescue can prevent extinction following environmental change. Ecol Lett.

[89] Lynch M, Lande R (1998). The critical effective size for a genetically secure population. Anim Conserv.

[90] Lehe R, Halltschek O, Peliti L (2012). The rate of beneficial mutations surfing on the wave of a range expansion. PLoS Comput Biol.

[91] Valero M, Destombe C, Mauger S, Ribout C, Engel RC, Daguin-Thiebaut C (2012). Using genetic tools for sustainable management of kelps: a literature review and the example of Laminaria digitata. Cah Biol.

[92] Allendorf FW, England PR, Luikart G, Ritchie PA, Ryman N (2008). Genetic effects of harvest on wild animal populations. Trends Ecol Evol.

[93] Tomanek L (2008). The importance of physiological limits in determining biogeographical range shifts due to global climate change: the heat-shock response. Physiol Biochem Zool.

[94] Hampe A, Petit RJ (2005). Conserving biodiversity under climate change: the rear edge matters. Ecol Lett.

[95] Diekmann OE, Serrão EA (2012). Range-edge genetic diversity: locally poor extant southern patches maintain a regionally diverse hotspot in the seagrass *Zostera marina*. Mol Ecol.

[96] Leclerc MC, Barriel V, Lecointre G, De Reviers B (1998). Low divergence in rDNA ITS sequences among five species of *Fucus* (Phaeophyceae) suggests a very recent radiation. J Mol Evol.

[97] Coyer JA, Hoarau G, Oudot-Le Secq MP, Stam WT, Olsen JL (2006). A mtDNA-based phylogeny of the brown algal genus *Fucus* (Heterokontophyta; Phaeophyta). Mol Phylogenet Evol.

[98] Cánovas FG, Mota CF, Serrão EA, Pearson GA (2011). Driving south: a multi-gene phylogeny of the brown algal family Fucaceae reveals relationships and recent drivers of a marine radiation. BMC Evol Biol.

[99] Nicastro KR, Zardi GI, Teixeira S, Neiva J, Serrão EA, Pearson GA (2013). Shift happens: trailing edge contraction associated with recent warming trends threatens a distinct genetic lineage in the marine macroalga *Fucus vesiculosus*. BMC Biol.

[100] Piñeiro-Corbeira C, Barreiro R, Cremades J (2016). Decadal changes in the distribution of common intertidal seaweeds in Galicia (NW Iberia). Mar Environ Res.

[101] Assis J, Berecibar E, Claro B, Alberto F, Reed D, Raimondi P (2017). Major shifts at the range edge of marine forests: the combined effects of climate changes and limited dispersal. Sci Rep.

[102] Smale DA, Wernberg T (2013). Extreme climatic event drives range contraction of a habitat-forming species. Proc R Soc B Biol Sci.

[103] Assis J, Coelho NC, Lamy T, Valero M, Alberto F, Serrão EÁ (2016). Deep reefs are climatic refugia for genetic diversity of marine forests. J Biogeogr.

[104] Pearson GA, Lago-Leston A, Mota C (2009). Frayed at the edges: selective pressure and adaptive response to abiotic stressors are mismatched in low diversity edge populations. J Ecol.

[105] Gaylord B, Reed DC, Washburn L, Raimondi PT (2004). Physical-biological coupling in spore dispersal of kelp forest macroalgae. J Mar Syst.

[106] King NG, McKeown NJ, Smale DA, Moore PJ. The importance of phenotypic plasticity and local adaptation in driving intraspecific variability in thermal niches of marine macrophytes. Ecography (Cop). 2017; 10.1111/ecog.03186. Blackwell Publishing Ltd

[107] Lourenço CR, Zardi GI, McQuaid CD, Serrão EA, Pearson GA, Jacinto R (2016). Upwelling areas as climate change refugia for the distribution and genetic diversity of a marine macroalga. J Biogeogr.

