# Supplementary figures and images for: Decadal stability in genetic variation and structure in the intertidal seaweed Fucus serratus (Heterokontophyta: Fucaceae)
Source: BMC Evol Biol. 2018 Jun 15;18:94. doi: 10.1186/s12862-018-1213-2 (PMC6002991; doi:10.1186/s12862-018-1213-2)

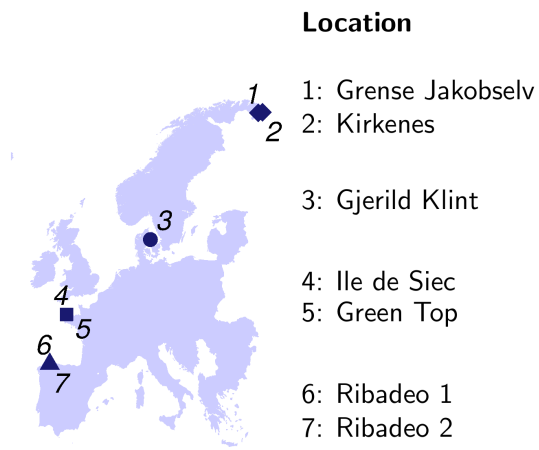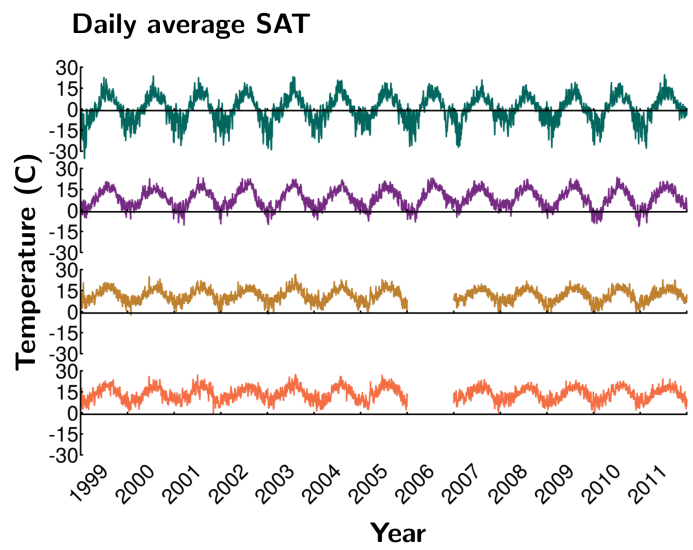

AdditionalFile 1

Supplement: Supplementary file 1 — Surface air temperatures. Daily average surface air temperatures (SAT) at each of the seven sampling sites from 1999 to 2011 with gaps in year 2006 for the French and Spanish sampling sites. SATs were identical between the two Norwegian sampling sites as well as between the two French and the two Spanish sampling sites. (PDF 513 kb) [file 12862_2018_1213_MOESM1_ESM.pdf]

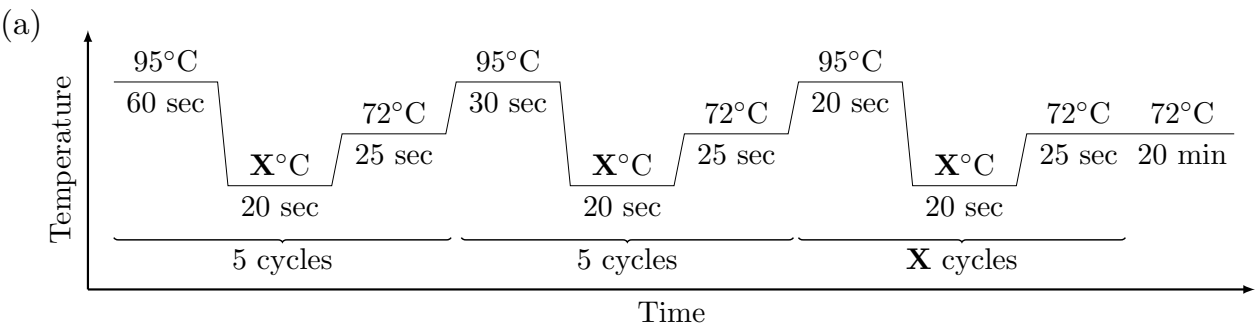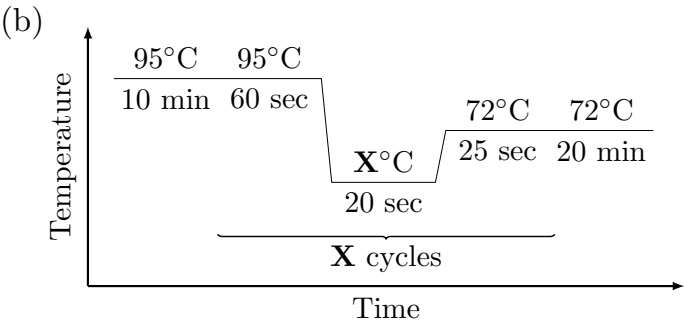

Supplement: Supplementary file 3 — PCR cycling protocols. Time-release (a) and no-time-release (b) PCR cycling protocols. In the time-release protocol, the heat-activated DNA-polymerase was progressively released during the thermal cycling process. Annealing temperatures and number of cycles indicated with an X are specified for each marker in Additional file 2. (PDF 34 kb) [file 12862_2018_1213_MOESM3_ESM.pdf]
